# What’s in the Box? Preschoolers Consider Ambiguity, Expected Value, and Information for Future Decisions in Explore-Exploit Tasks

**DOI:** 10.1162/opmi_a_00110

**Published:** 2023-10-27

**Authors:** Elizabeth Lapidow, Elizabeth Bonawitz

**Affiliations:** Department of Psychology, University of Waterloo, Waterloo, ON, Canada; Graduate School of Education, Harvard University, Cambridge, MA, USA

**Keywords:** exploration, explore-exploit, decision-making, cognitive development, uncertainty, ambiguity, expected value, information gain

## Abstract

Self-directed exploration in childhood appears driven by a desire to resolve uncertainties in order to learn more about the world. However, in adult decision-making, the choice to explore new information rather than exploit what is already known takes many factors beyond uncertainty (such as expected utilities and costs) into account. The evidence for whether young children are sensitive to complex, contextual factors in making exploration decisions is limited and mixed. Here, we investigate whether modifying uncertain options influences explore-exploit behavior in preschool-aged children (48–68 months). Over the course of three experiments, we manipulate uncertain options’ ambiguity, expected value, and potential to improve epistemic state for future exploration in a novel forced-choice design. We find evidence that young children are influenced by each of these factors, suggesting that early, self-directed exploration involves sophisticated, context-sensitive decision-making under uncertainty.

## INTRODUCTION

Exploration is a quintessential characteristic of childhood and early learning. Children’s self-directed engagement with their environment during exploratory play is central to theories of cognitive development (e.g., Bruner et al., 1976; Golinkoff et al., 2006; Piaget, 1930), and empirical evidence shows that this engagement develops early. Infants and toddlers selectively allocate attention to events with the highest informational content (Kidd et al., 2012, 2014), show sensitivity to events that will disambiguate confounded variables (Begus & Bonawitz, 2020), and preferentially engage with what is unexpected or belief-violating during their play (Baldwin et al., 1993; Stahl & Feigenson, 2015). From the first, self-directed exploration seems aimed at improving and expanding knowledge. However, choosing whether and when to explore is a complex decision involving multiple factors, particularly when choosing to seek new information means forgoing an opportunity to exploit a known reward. The current study aims to examine young children’s sensitivity to several complex factors that are relevant to such decisions.

Past theories of children’s exploration decision-making have focused largely on the role of *uncertainty*. Early work (e.g., Weisler & McCall, 1976) distinguished exploration from other self-directed behaviors as actions taken to reduce current uncertainty or acquire new information. More recently, Theory Theory accounts of cognitive development suggest that uncertainty between prior beliefs and current evidence plays a key role in exploration: When faced with ambiguous or conflicting hypotheses, learners are motivated to seek new information in order to resolve the uncertainty between them (e.g., Gopnik & Wellman, 2012; Schulz, 2012). There is considerable empirical evidence for this account. In studies of free-play, children preferentially explore where the immediate evidence is most uncertain. That is, young learners seem motivated to investigate and interact with elements of the environment about which their current knowledge is incomplete (e.g., Kidd et al., 2012; Liquin & Lombrozo, 2020; Siegel et al., 2021), ambiguous (Cook et al., 2011; Gweon & Schulz, 2008; Schulz & Bonawitz, 2007), or inconsistent with prior beliefs (Bonawitz et al., 2012; Schulz et al., 2008; van Schijndel et al., 2015). There is also recent evidence to suggest that preschoolers have an early-developing intuitive sensitivity to the uncertainty of their own beliefs and preferentially test or reveal information that can improve their epistemic position (Lapidow, Killeen, & Walker, 2022; Wang et al., 2021).

However, while young children seem motivated to explore uncertain outcomes, there is also evidence to suggest that these decisions may not be systematic, selective, or sensitive to complex factors beyond uncertainty. This is particularly true of *explore-exploit* contexts, in which agents must adaptively trade-off between gathering new information by revealing uncertain reward outcomes or gaining known rewards from certain reward outcomes (see Wilson et al., 2021 for review). Within these tasks, an agent might explore either with a goal or policy in mind (‘directed exploration’) or more randomly, and recent research suggests that the kind of exploration learners tend to engage in changes with age: Random exploration is highest in preschoolers and decreases into adulthood (Meder et al., 2021; Plate et al., 2018; Schulz et al., 2019). These studies also find that even when children engage in directed exploration, they often pursue uncertainty when it is not the optimal strategy. Consistent with this, both adults’ and 4- to 9-year-olds’ self-reported curiosity to learn outcomes in multi-armed bandit tasks follows expected learning, but children show an additional sensitivity to uncertainty that adults do not (Liquin et al., 2021).

Importantly, while the *explore-exploit* dilemma fundamentally characterizes exploration as a choice to reveal uncertain outcomes (e.g., Frank et al., 2009; Gittins & Jones, 1974), adaptive decisions within these contexts must consider more than uncertainty alone. Research with adults indicates a complex consideration process, in which the costs of seeking out new information trades-off against the utility of gaining that information (e.g., Fu & Gray, 2006; Gigerenzer et al., 2012; Newell & Shanks, 2003) and in which environmental, personal, and social factors all have an influence on decision-making (see Mehlhorn et al., 2015 for review). For example, adults sample more information, even at the cost of money and time, prior to making consequential choices than they do in situations with lower stakes (Juni et al., 2016). This is a very different perspective on exploration under uncertainty, one that includes sensitivity to and consideration of additional situational factors, than is typically taken in research on exploration in childhood. Indeed, almost all of the research on early exploration described above focuses on comparing children’s behavior in the presence of uncertainty to its absence. In contrast, research on mature explore-exploit decisions focuses on how the manipulation of *other* factors—such as the efficiency of information (Meier & Blair, 2013), stability of the environment (Knox et al., 2012), time horizons for exploration (Somerville et al., 2017), expectations about rare events (Teodorescu & Erev, 2014), and so on—lead to differences in adults’ decision-making.

### Situational Factors in Early Exploration Decisions

From infancy onwards, children recognize and respond to uncertainty with exploration, but may not engage in more complex consideration of uncertainties until much older. Here, we examine this possibility by investigating whether and how sensitivity to complex situational factors influences decisions to explore uncertainties in early childhood. Specifically, we will look at preschooler’s decisions to explore uncertainties in the presence of ambiguity (Experiment 1), differences in expected values (Experiment 2), and different potentials for gaining information for future decision making (Experiment 3). These are, of course, by no means the only factors that influence mature explore-exploit decision-making. The choice to focus on these three factors in this initial investigation was motivated by the extensive evidence of their prominent role in adult decision-making and the mixed evidence for children’s sensitivity to them. Below, we briefly review the past research on each of these three factors before introducing the overall design of the current investigation.

#### Factor One: Ambiguity.

One important nuance in consideration of uncertain outcomes is the source of its uncertainty, that is, the level at which the learner is missing information. Learners can have uncertainty about a specific outcome, which occurs when outcomes are probabilistically determined by a known distribution. However, in some cases, learners may also have uncertainty *about* the distribution itself. The term *ambiguity* captures these cases in which agents are missing the information necessary for the prediction of an outcome (Frisch & Baron, 1988).

A classic example of ambiguity and how it is distinct from uncertainty comes from Ellsberg (1961) urn problem. Two urns (*A* and *B*) are each filled with 100 balls. Urn *A* contains 50 red balls and 50 black balls, while urn *B* contains an unknown ratio of red and black. One concealed ball is drawn at random from each urn and participants choose which outcome (‘*Is the drawn ball red or black?*’) to gamble on. For both urns, this outcome is uncertain; however, in urn *A* the probability of each possible outcome is known. In contrast, the participant has no information about the probability of the possible outcomes for a ball drawn from urn B, making that outcome *ambiguous*. Ellsberg (1961) found that people overwhelmingly preferred to bet on the outcome of balls drawn from urn *A*, rather than those from urn *B*.

Extensive subsequent investigation of ambiguity with adults has consistently and robustly replicated this tendency for *ambiguity aversion* (see Camerer & Weber, 1992; Trautmann & van de Kuilen, 2015 for reviews). Across a variety of task designs, adult decision-makers almost always prefer uncertain options over ambiguous ones. Even in contexts where knowing the probabilities does not confer any substantial advantage to decision-makers (such as with the 50/50 probabilities of the urn problem), adults are highly resistant to selecting options for which they lack this knowledge (e.g., Becker & Brownson, 1964; Heath & Tversky, 1991; Lauriola & Levin, 2001, etc.).

Given the significance of ambiguity to adults’ decision-making under uncertainty, our first experiment investigates if and how this factor might influence children’s exploration and exploitation. From the existing research, it is not clear whether young children even distinguish ambiguity from uncertainty in paradigms with two exploratory options, let alone how exploration of ambiguity trades off with exploiting known outcomes. Studies on explore-exploit behavior have found that 8- to 9-year-olds are less ambiguity averse than adults (Li et al., 2015) and that aversion increases linearly with age between 10- and 25-years-old (Blankenstein et al., 2016). One study has looked at exploratory choices between ambiguous and uncertain options in preschoolers: Li et al. (2017) found that although 5-year-olds exhibited consistent choice behavior, there was no evidence of ambiguity aversion. In fact, in cases where the uncertain option’s known probabilities indicated even odds of winning or losing (as in the 50/50 urn problem), children chose at chance between this and an ambiguous alternative. While these results suggest that preschoolers are not sensitive to the difference between ambiguity and uncertainty, it does not shed light on whether children’s explore-exploit behavior differs depending on whether or not the uncertainties available to explore are ambiguous. In addition, since both the options in Li et al. (2017) were uncertain, children’s lack of sensitivity may have been due, at least in part, to cognitive limitations in maintaining multiple uncertain representations simultaneously. Thus, it remains an open question how preschool-aged children navigate the trade-off between exploitation and ambiguous exploration.

#### Factor Two: Expected Value.

When learners *do* have knowledge about the overall distribution of an uncertain outcome, it both removes ambiguity and allows for consideration of expected value. The expected value of an uncertainty (i.e., the sum of the values of each of the option’s possible outcomes, weighted in proportion to their probability of occurring), is foundational in decision theory accounts of how agents understand and operate within an uncertain world (for reviews, see Feather, 1982; Wright, 1984). Furthermore, in adults, the decision to switch from exploiting known rewards to exploring uncertainties seems to hinge on consideration and comparison of the expected value, the potential costs and gains, of each (Mehlhorn et al., 2015).

While there is no doubt that young children are aware of probability information (e.g., Denison et al., 2006; Gweon et al., 2010; Kushnir & Gopnik, 2005; Xu & Garcia, 2008, etc.), and can even use it to predict likely outcomes of uncertain options (Denison & Xu, 2010, 2014; Lapidow et al., 2021; Lapidow, Goddu, & Walker, 2022), their ability to utilize estimates of expected values from probability information in explore-exploit decision-making has been under-investigated. Encouragingly, 4- and 5-year-olds appear to have an intuitive grasp of expected value when it comes to *evaluating* uncertainties. When asked to judge the goodness of uncertain options, children appropriately weight an outcome’s intrinsic reward amount by the probability of this outcome occurring (Anderson, 1980; Bayless & Schlottmann, 2010; Schlottmann, 2001; Schlottmann & Anderson, 1994; Schlottmann & Tring, 2005). They can even integrate the probability of a reward with the expected cost (difficulty) of completing an action (Wang & Bonawitz, 2022).

However, this previous research also finds that young children struggle to form accurate expected values for options with multiple non-zero possible outcomes (e.g., Schlottmann, 2001). Although this difficulty is not unique to children’s decision-making (see Shanteau, 1975), adults consistently outperform 5- to 10-year-olds in evaluating expected-values that require integrating over multiple outcomes (Schlottmann, 2000). Furthermore, when asked to make a *choice* rather than an evaluation, preschoolers often fail to utilize probability information in their decision making (e.g., Betsch & Lang, 2013; Garon & Moore, 2004; Huizenga et al., 2007). For example, Levin et al. (2007) gave 5- to 11-year-olds and adults the choice between two options with two, three, or five possible outcomes each. One of these options was ‘certain’, since all the possible outcomes contained the same reward amount. For the ‘uncertain’ option, one outcome also contained this amount, but all other outcomes contained zero reward. By manipulating the number of possible outcomes and the reward amounts, the authors varied the relative expected value of the certain and uncertain options over different trials. The choice behavior of 5- to 7-year-olds showed very little response to these changes, significantly less than older children (8- to 11-years-old) and adults.

Thus, despite the foundational role of expected value to uncertain decision-making, there is reason to doubt whether preschoolers will make accurate use of probability information in determining when to explore versus exploit. However, the design of previous tasks may have made it artificially difficult for preschoolers to track and compare possible rewards. In Levin et al. (2007) in particular, the need to integrate over multiple possibilities for the *certain* option may have been especially challenging and made the difference from the uncertain option unclear to younger children. In the current task, therefore, we examine this ability in a design that only requires children to consider multiple possible outcomes for the exploration option, while the exploitation option has a single fixed outcome.

#### Factor Three: Information Gain.

Revealing uncertain outcomes, by definition, provides a learner with previously unknown information (Lindley, 1956; Shannon, 1948). This reality is the basis for Theory Theory’s suggestion that spontaneous exploration behavior is guided by a motivation to resolve uncertainties in order to improve and expand their current knowledge. However, new information does not necessarily equate to new knowledge. For example, the outcome of a concealed die roll is uncertain, but if its weighting is already known, or the learner does not expect this die to be used again for future rolls, then revealing the outcome does not usefully increase their knowledge. In order for exploration to support learning, making successful choices about what to explore requires evaluating not only *whether* there is uncertainty to be reduced, but also the potential for reducing it to meaningfully improve one’s current knowledge.

Sensitivity to the expected future value of new information is regularly observed in adult explore-exploit decision-making. For example, adults engage in more exploration, even at cost, when revealing uncertainties provides information relevant to a subsequent high-stakes decision (Juni et al., 2016). More generally, the importance of this factor to adult decision making is indicated by the influence of *task horizons*, how many future choices the decision-maker believes are before them. Importantly, length of task horizon has no effect on the actual amount of reward obtained from an uncertain option, but rather reflects the increase in the value of seeking new information: The longer the horizon (the more future encounters with the uncertainty environment are expected), the greater the value of choosing to explore uncertainties (Rich & Gureckis, 2018). Adults consistently make more exploratory choices when the horizon is longer (Lee et al., 2011; Meyer & Shi, 1995; Wilson et al., 2014), even when the exact length of the horizon is itself uncertain (Rich & Gureckis, 2014, 2018).

Past research offers mixed suggestions about whether or not preschoolers are likely to track the potential utility of information in their explore-exploit decisions. Recent research suggest that young children are sensitive to the utility of information in their exploration decisions: Both Blanco and Sloutsky (2021) and Meder et al. (2021) find that children as young as 4-years-old actively seek information in their explore-exploit decisions in a way that accurately tracks uncertainty reduction. Similarly, outside of explore-exploit tasks, children’s attention to uncertain outcomes seems strongly informed by expectations about the potential to support their learning: children’s curiosity about uncertain outcomes is predicted by expected learning (Liquin et al., 2021) and they choose to reveal uncertain information in domains where they hold competing theories, rather than mature knowledge (Wang et al., 2021).

On the other hand, children are often reported to be indiscriminate and inefficient in their exploration choices even after the preschool years. Somerville et al. (2017) find evidence that sensitivity to time horizons in explore-exploit paradigms emerges during adolescence, rather than in childhood. More recently, Zhuang et al. (2023) compared 5- to 6-year-olds, 11- to 12-year-olds, and adults on an explore-exploit task under short, long, and ambiguous time horizons and found that adaption to these differences depended on, and increased with, age. While older children’s explore-exploit behavior changed in response to task horizons in the same way as adults, younger children’s behavior did not show evidence of sensitivity to these manipulations. This more protracted development is also suggested by research on the development of information search more broadly. Gradeschoolers (7- to 10-year-olds) are more likely than adults to continue seeking information after no ambiguity remains (e.g., Davidson, 1991; Ruggeri et al., 2016) and are less attuned to expected learning in their choice of interventions (Nussenbaum et al., 2020). Such behavior is consistent with an exploration decision process that is driven and directed primarily by the *presence* of uncertainty, rather than by the potential for revealing uncertain outcomes to improve current knowledge.

Experiment 3 aims to help adjudicate between these findings by investigating whether children’s decisions to explore are sensitive to whether or not the information revealed by exploration will be useful for later decision-making. Notably, such a comparison is offered by studies of free-play (Bonawitz et al., 2012; Cook et al., 2011; Stahl & Feigenson, 2015, etc.) in which children are given evidence that is either complete and certain (thus, further exploration will not likely lead to increased knowledge) or ambiguous and incomplete (thus, there is something to be learned by exploration) and then allowed to explore. In contrast, the existing research on children’s explore-exploit behavior has not directly compared behavior in contexts where revealing uncertainties offers the potential for learning to contexts in which it does not. Here, we make an attempt to narrow the gap between this exploration research and explore-exploit research by asking whether an expectation that revealing uncertainties will or will not increase their knowledge influences how often children choose to explore.

### The Current Study

The goal of the current study is to shed light on the development of explore-exploit behavior by investigating whether three situational factors prominent in adult decision-making also influence the decision to explore uncertainties in early childhood. To do this, we designed a task in which participants attempt to collect marbles to use in a marble-maze toy by selecting paper boxes that have different amounts of marbles indicated inside. On each task trial, participants are offered the choice to collect marbles from one of two boxes: a *known box*, which is open to show the amount inside, and an *unknown box*, which is drawn at random from a set of identical, closed boxes. Thus, children are repeatedly presented with a choice either to *explore* by selecting (and then getting to open) the unknown box, or to *exploit* by selecting the known box. In order to examine the three factors of interest, we manipulate what participants do or do not know about the distribution of marble amounts in the boxes from which the unknown box is drawn and whether it is possible to learn this distribution through exploration. If young children’s exploration decisions are determined by a simple preference for uncertainty, then we would expect participants to consistently select the unknown box, regardless of other manipulations. If, however, young exploratory learners are sensitive to any of the situational factors manipulated, then we would instead expect to see different patterns of decision-making in each of the three experiments. By determining whether children’s self-directed exploration decisions are sensitive to complex, contextual factors this work aims to help provide a more accurate understanding of the exploration behavior that characterizes early childhood.

## EXPERIMENT 1: AMBIGUITY

Ambiguity, situations in which agents have no information about the possible outcomes of an uncertainty, has a clear influence on adult decision-making. However, past research suggests that young children may not be sensitive to ambiguous contexts when deciding between exploration and exploitation. Notably, this would not be entirely inconsistent with the preference for exploring novel and unknown outcomes that is characteristic of childhood and documented in previous exploration research. If early learning is supported by a generalized tendency to reveal and resolve uncertainties in the environment, a lack of ambiguity aversion broadens the space of contexts in which children are comfortable engaging in learning.

Our first experiment presents a forced-choice between explore-exploit alternatives in which the outcomes of exploration options were entirely ambiguous. Participants had no prior information about the distribution of rewards in the closed boxes and thus could not infer the possible outcomes of exploring uncertainties. If ambiguity is not a factor in children’s exploration decisions, then this absence of probability information should not have an impact on their choice behavior. In this case, we would expect to see children preferentially selecting the unknown box, consistent with the preference for uncertainty seen in previous exploration research. Alternatively, if young learners have a similar sensitivity to ambiguity as adult decision-makers, then we would expect them to avoid these options by choosing to exploit the known alternative on the majority of task trials.

### Method

#### Participants.

Eighteen preschoolers (44.4% female, *M* = 54.47 months, *SD* = 4.5 months, Range = 48–63 months) participated in Experiment 1. Children were recruited from and tested in local schools and activity centers (zoo, museums, etc.) in the Newark, New Jersey area and were approximately representative of the local population. Four children were dropped and replaced for failing to answer the practice question correctly.

#### Materials.

**Figure 1.**
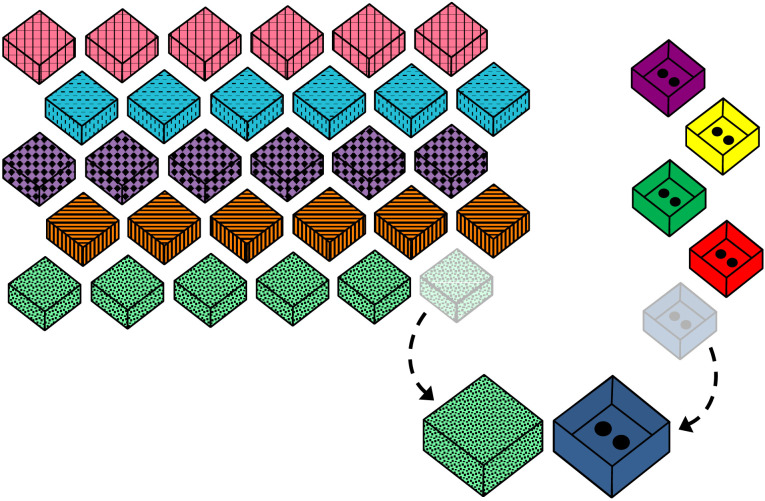
Setup of stimuli at the end of the first task trial of Experiment 1. *Note*. The order of the unknown sets (left-hand side) was counterbalanced across participants, while the paired known boxes (right-hand side) was the same for all participants.

The task was conducted using a total of 37 boxes made from folded paper. Two of these were made of white paper (‘practice boxes’): one with one dot and one with three dots drawn on the inside of the lid. The remaining 35 boxes had a variety of colors and patterns on the outside and different numbers of dots concealed inside. Of these, the 30 ‘*unknown boxes*’ were organized into five ‘sets’ of six boxes each. All the boxes in an unknown set were identical in appearance, and each set was visually distinct from the other four (Figure 1). The remaining five boxes were each entirely unique in appearance and had two dots drawn inside. Each of these five ‘*known boxes*’ was paired with one of the five sets, and these pairings were consistently used across all participants. A commercially available marble maze toy and marbles were also used. Lists and images of the materials, procedure scripts and data are all available on OSF at: https://osf.io/cf87b/?view_only=555e65fef7c54093b53410e1d379a17b.

#### Procedure.

At the start of the session, the five sets of unknown boxes were arranged in rows on the table in order of presentation. The five known boxes and two practice boxes were placed out of sight of the participant. The maze toy and marbles were placed in a visible location well out of reach of participants.

The experimenter told children that they were going to play a game using the boxes. She explained that the boxes could have different numbers of dots drawn inside them and that the child would receive one marble for every dot inside the boxes they picked during the game. The experimenter then pointed out the marble maze toy and told children that they would get to put any marbles they got from the boxes into the maze at the end of the game.

To ensure that children fully understood the goal of the task and had the necessary numerical understanding, the experimenter then held out the two practice boxes and showed their contents (one and three dots, respectively) to participants. She asked: “Between these two boxes, which one would you pick to get *more* marbles?” Only children who correctly selected the box with three, rather than one dot, were included in the study (those who failed were allowed to play briefly with the maze toy, but did not complete any task trials).

Following this check, five task trials were administered (order of trials counterbalanced across participants). Figure 1 shows the layout of stimuli at the end of the first trial of Experiment 1. On each trial, the experimenter moved the next set of unknown boxes from the rows on the table in front of the child and selected one at random. Holding this box closed in one hand, the experimenter would then bring out the known box for that set. The experimenter opened this box and held it in her other hand. Children were then offered a choice between getting marbles from the known box (“where we know there are two inside”) or the unknown box (“where we don’t know how much is inside”). The order in which these two options were named and which hand held which box was alternated across task trials. After children made their choice, the experimenter set the chosen box to one side, explaining that they would fill it with marbles (and, if the unknown box was chosen, open it and find out how many dots were inside) at the end of the game. Both the unselected option and the remaining five boxes from the unknown set were removed out of the participant’s view, after which the experimenter would begin the next trial with the next set of boxes.

After completing all task trials, the experimenter moved the five boxes selected by the participant back to the center of the table. All the boxes were then opened to reveal the dots inside, and the experimenter placed the matching number of marbles into each one. Children were allowed to put each of these marbles into the maze toy as a reward for participating in the task.

### Results and Discussion

Participants’ choices on each trial of the task were recorded and scored for the number of times the unknown box was selected. Overall, children chose to explore on 32 out of 90 trials. In order to account for possible effects of individual and trial order, we fitted a constant (intercept-only) logistic mixed-effects model to look at children’s choices to explore the unknown box, including random intercepts for each participant and for trial order. The model’s intercept estimate for the probability of choosing to explore was 0.323 (95% CI [0.176, 0.515], *p* = 0.07) and the estimated random effects were 0.96 for participant and 0.23 for trial order, indicating substantial between-subjects variance.

When presented with the choice to explore in an ambiguous context, preschoolers explored on only 35.56% of trials, more often choosing to instead avoid uncertainty and exploit options with known outcomes. That this was only marginally different from chance, coupled with the random effect of participant, suggests individual differences in how ambiguity influenced children’s exploration decisions. This result immediately suggests that only considering the presence or absence of uncertainty is insufficient for a complete account of exploration decisions in early childhood. Despite children’s well-documented preference for exploring what is unknown, uncertain, and novel in their surroundings, the opportunity to explore in the current task was unappealing to many children. These findings suggest that young children, like adults, are sensitive to and tend to avoid ambiguity when choosing whether to explore and further that the influence of this sensitivity may differ between individuals. Of course, it is also possible that exploring uncertainty in the current task was simply unappealing or discouraged by a different factor than ambiguity. These possibilities are ruled out by performance in Experiment 2.

Importantly, however, this result is not evidence of *classic* ambiguity aversion, which involves a choice between ambiguous and uncertain options, rather than between ambiguous and known options. The difference between a gamble about which you know nothing and a non-gamble might be easier for young children to track than the difference between two gambles for which your amount of knowledge differs. This would explain the difference between the current results and those of Li et al. (2017). An alternative possibility is that children had a hyperprior that the expected value of the unknown boxes would be less than the two marbles offered by the exploitation option (despite the initial inclusion criteria comparing 1 to 3 marbles that could have set this expectation to 2). Experiment 2 partially rules out this alternative. The results of Experiment 1 show that children as young as four *recognize* when an uncertainty is ambiguous and, as we will investigate further in Experiment 2, may treat this differently in their exploration decision-making than the presence of uncertainty alone.

## EXPERIMENT 2: EXPECTED VALUE

The results of Experiment 1 suggest that, like adults, preschoolers had a tendency to exploit known rewards when there was ambiguity about the expected outcome of exploring. In Experiment 2, we turn to our second question: When there *is* knowledge about the overall distribution of an uncertain outcome, can children use the expected value of this distribution to inform their explore-exploit decisions? Specifically, we ask whether presenting preschoolers with information about the possible reward values of the unknown box influences their explore-exploit decisions in a coherent way.

The procedure closely follows that of Experiment 1, except that participants are shown the contents of each of the boxes in a set, after which all the boxes are closed, shuffled, and one is selected at random as the unknown option. While the actual amount of reward in this box is unknown, this information allows children to construct an estimate of the expected value of its contents (children are also given a picture of the set’s contents, so that this estimate does not need to be constructed from memory). Each set of boxes has a different distribution of rewards (i.e., the number of dots), and the expected values for the unknown box range from four times more or less than that of the known box. Table 1 shows the exact distributions and expected values for each of the sets used in the task along with the corresponding known amount. Note that, in all of these sets, the range of values always includes amounts above and below the certain value of the known exploitive option, so that simple heuristic cannot be used to guide decision-making.

**Table 1.** The individual values for individual boxes in each set of unknown boxes used in Experiment 2.

Ratio	Set A	Set B	Expected Value
**4:1**	0 - 0 - 0 - 0 - 0 - 0 - 0 - 4	0 - 0 - 0 - 0 - 0 - 0 - 1 - 3	0.5
**2:1**	0 - 0 - 1 - 1 - 1 - 1 - 1 - 3	0 - 0 - 0 - 0 - 1 - 1 - 1 - 5	1
**1:1**	0 - 1 - 1 - 1 - 3 - 3 - 3 - 4	0 - 0 - 0 - 0 - 3 - 4 - 4 - 5	2
**1:2**	1 - 4 - 4 - 4 - 4 - 5 - 5 - 5	0 - 0 - 5 - 5 - 5 - 5 - 6 - 6	4
**1:4**	1 - 8 - 8 - 8 - 9 - 10 - 10 - 10	0 - 9 - 9 - 9 - 9 - 9 - 9 - 10	8

*Note*. There were two sets of unknown boxes (A and B) for each of the five ratios.

If children do indeed consider the expected value of uncertainties in deciding whether or not to explore, then we would expect their choice behavior to follow *probability matching*. That is, the proportion of choices to explore the unknown box on any given trial should reflect the desirability of the unknown box’s expected value relative to the certain value of the known box (see Bonawitz, Denison, Griffiths, & Gopnik, 2014 for empirical support and an explanation of when and why children may probability match over “maximize” in these kinds of tasks). By contrast, if expected value does not influence exploration decisions, we would expect no relationship between choice behavior and the ratio of known to unknown values, with children favoring either the explore or the exploit option across all trials. It is also possible that performance on this task might reflect a mix of factors. That is, preschoolers may show some sensitivity to expected value while still under- or over-exploring on trials when the expected value of the unknown box is equal to that of the known box.

In addition to gauging the influence of expected value on children’s explore-exploit decisions, we will also look at how performance on this task compares to performance on Experiment 1. If the pattern of choice behavior in the first task were indeed a reflection of children’s tendency to avoid ambiguous exploration options, then we would expect to see more explore choices when that ambiguity is removed in Experiment 2.

### Method

#### Participants.

Twenty-four preschoolers (45.8% female, *M* = 59 months, *SD* = 5.2 months, Range = 48–66 months) participated in Experiment 2. Children were recruited from the same locations as in Experiment 1, but none of the children had participated previously. Two children were dropped and replaced, one for refusing to complete the testing session and one for failing to answer the practice question correctly.

#### Materials.

The stimuli included the same maze toy, marbles, and practice boxes as used in Experiment 1. A total of 90 new paper boxes were also constructed for this task: Ten unknown box sets, each containing eight identical boxes, and ten unique known boxes that were paired with each set. In addition, color photographs of each unknown set with their contents visible were used as reminders during the game.

#### Procedure.

**Figure 2.**
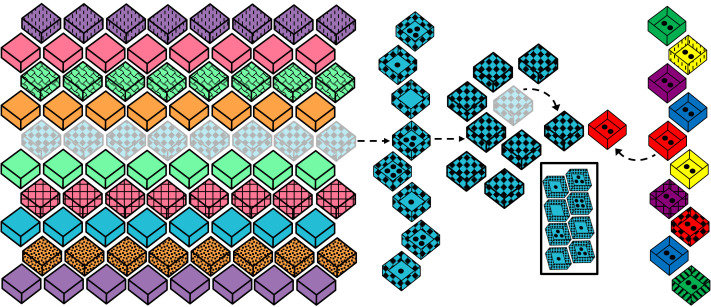
Setup of stimuli at the end of a single task trial in Experiment 2.

The set up, introduction, and practice question were all identical to Experiment 1. The ten task trials were also conducted in a similar manner, save for the inclusion of distribution information. At the start of every task trial, after the experimenter brought the next set of unknown boxes to the center of the table, she opened each box and counted out the number of dots inside each individual box with the participant. Simple encouragement statements and questions were used throughout the task to ensure children were equally engaged and attending to the contents of the boxes across all ten task trials. In order to avoid any interference from working memory, a color photograph of the set was brought out and shown to participants after counting was completed (Figure 2). The experimenter directed children to notice how the image matched the set they had just seen and explained that they should use the picture to help them remember what was inside the boxes. This image remained on the table in view of the participant until the end of the trial.

The experimenter then closed all eight of the boxes in the set and shuffled their positions. After the set was mixed sufficiently to ensure that individual boxes could not be visually tracked^1^, one box was selected at random to become the unknown option (see Figure 2). Presentation of the known box and final choice question were identical to Experiment 1. The only difference was that the experimenter described the unknown box as: “the box where we don’t know how much is inside, but we know it has to be one of these,” while indicating the reminder picture.

### Results and Discussion

Children in Experiment 2 chose to explore the unknown box on 139 out of the 240 total task trials. This was significantly more exploration (57.9% of choices) than in Experiment 1 (35.6% of choices), (*p* < 0.0001, two-tailed binomial), suggesting that the absence of ambiguity in this task had a substantial impact on increasing children’s exploration of uncertain options. This difference in choice behavior supports the tentative interpretation offered for Experiment 1: that the context of ambiguity in that task made the opportunity to explore less appealing for many children. The comparison makes it clear that children’s explore-exploit decisions are sensitive to the presence of information about the possible outcomes of uncertain options. In order to understand *how* this sensitivity influenced their decision process, we fitted a logistic mixed model to predict choosing the unknown box with expected value, including random intercepts for participant and trial order. The model indicated a substantial random effect of participant (6.3) and no effect of trial order (0.0). A likelihood ratio test using the *lrtest* package revealed a significant overall effect of expected value on choices to explore when this model was compared to a null model with random effects of participant and trial order, X2(−4) = 14.62, *p* < 0.01.

**Figure 3.**
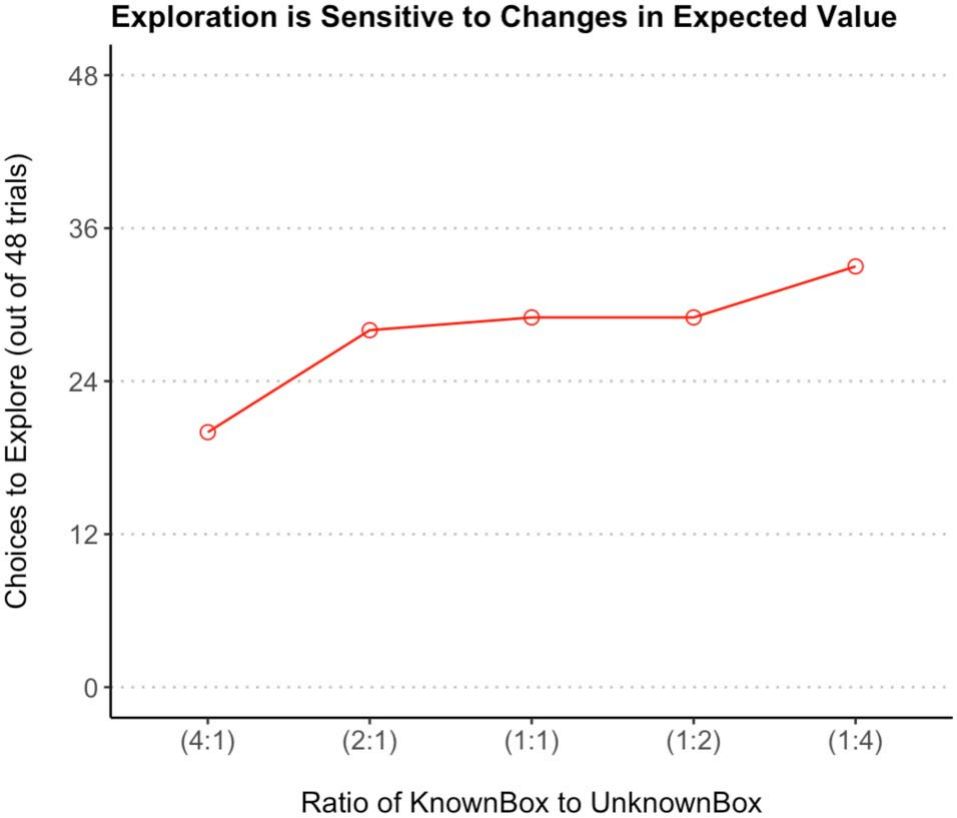
Relationship between children’s choices to explore and the ratio of known to unknown reward amounts in Experiment 2. *Note*. Error bars represent standard error.

We also looked at children’s performance on the different trials (i.e., at each of the five different expected values for the unknown option). Overall, as the expected value of the unknown option increased relative to the known, so did children’s choices to explore (Figure 3). We fitted a logistic mixed-effects model predicting choice to explore with expected value (treated as a factor with five levels) and controlling for random effects of individual and trial order. The probability of choosing to explore estimated for each expected value was significantly different from chance: 0.32 for an expected value of 0.5 (or a 4:1 ratio of known option value to expected value), 0.63 for an expected value of 1 (2:1 ratio), 0.67 for an expected value of 2 (1:1 ratio), 0.67 for expected value of 4 (1:2 ratio), and 0.81 for expected value of 8 (1:4 ratio), all *p* < 0.001. Using the *emmeans* package, we conducted post-hoc pairwise comparisons of the different trials, with Tukey corrections for multiple comparisons. All but one of these contrasts was significant (see Table 2). Exploration on trials with a 4:1 or 2:1 known to expected value ratio was significantly lower than on all other trials, and exploration on trials with a 1:4 ratio was significantly higher than on all other trials (all *p* < 0.001). Unsurprisingly, given that they had the same probability of choosing to explore, the 1:1 and 1:2 ratio trials did not differ (*p* = 1).

**Table 2.** Pairwise comparison of differences in exploration between box sets in Experiment 2.

Comparison	Estimated Odds Ratio (*SE*)	*z*-ratio	*p*-value
**4:1** vs. **2:1**	0.27 (0.0004)	−921.41	*< 0.0001*
**4:1** vs. **1:1**	0.23 (0.0004)	−1040.37	*< 0.0001*
**4:1** vs. **1:2**	0.23 (0.0004)	−1040.23	*< 0.0001*
**4:1** vs. **1:4**	0.12 (0.0002)	−1554.77	*< 0.0001*
**2:1** vs. **1:1**	0.85 (0.0017)	−84.15	*< 0.0001*
**2:1** vs. **1:2**	0.85 (0.0017)	−84.04	*< 0.0001*
**2:1** vs. **1:4**	0.41 (0.0008)	−448.10	*< 0.0001*
**1:1** vs. **1:2**	1.00 (0.002)	0.106	1.0000
**1:1** vs. **1:4**	0.48 (0.001)	−363.97	*< 0.0001*
**1:2** vs. **1:4**	0.48 (0.001)	−364.07	*< 0.0001*

As a final analysis approach, we also built generative probabilistic binomial models of responding, allowing us to directly contrast the likelihood of the children’s responses under each “coin-flipping” model. Doing so provides quantitative exploration of the processes (i.e., sensitivity to expected value, bias toward exploration) that best explain children’s responses. The binomial models treat each response as an independent Bernoulli process. Our models allowed for different probabilities, p, for each trial type (somewhat like “slopes” in a linear representation) and set an overall exploration “weight” (somewhat like an “intercept” in a linear representation). The first model we built captures a case where children are simply randomly guessing/indifferent to trial type for all responses (“random guessing, no bias”: p = .5 for all trials). A second model captures cases where children are still not sensitive to trial type, but allow for a mild exploratory bias; we set this “bias” given the overall exploration average of the children’s responses across trial types which was .079 percentage points higher than random responding, (“random guessing, explore bias”: p = .579 for all trial types). The next model reflected sensitivity to trial type, with the p set by the expected value given in the experimental condition, but with no exploratory bias (“EV sensitive, no bias”: e.g., a 1:4 condition would have p = .200, in 1:2 condition p = .333, in 1:1 condition p = .500, etc.). The fourth and final model included both sensitivity to trial type and exploratory bias. For this model, p was computed as an additive mixture of the trial type given by the expected values and the constant exploration bias, (“EV sensitive, explore bias”: e.g., 1:4 condition would have p = (.2 + .079) = .279, 1:2 condition p = (.333 + .079) = .412, 1:1 condition p = (.5 + .079) = .579, etc). Using these contrastive binomial models, we computed log-likelihood scores for the children’s responses. The likelihood scores were similar across the first three models (random guessing, no bias: −71.0; random guessing, explore, bias: −72.2; EV sensitive, no bias: −75.9) but much larger for the model with mild exploratory bias and probability set by the expected values (EV sensitive, explore bias: −58.5). Together, the results of Experiment 2 suggest that preschoolers are not only sensitive to the presence of probability information about uncertain outcomes, but also effectively utilize this information to form expected values for these options when determining whether to explore. They also indicate the possibility of a mild exploratory bias in tasks where probability information is present.

Children’s behavior in Experiment 2 also supports recent probability matching, rational learning accounts. The approximately linear trajectory of children’s exploratory preference by condition on this task is consistent with probability matching. There is a large literature demonstrating children’s tendency to probability match, rather than maximize, on probabilistic sampling tasks like Experiment 2 (e.g., Bonawitz, Denison, Gopnik, & Griffiths, 2014; Bonawitz, Denison, Griffiths, & Gopnik, 2014; Denison et al., 2013). This research suggests that probability matching behavior indicates children’s engagement in an implicit statistical sampling of outcomes as potential responses rather than an exhaustive evaluation of options and computing of an optimal response which would lead to maximizing. They further argue that a tendency towards sampling and probability matching has a rational basis for young learners; optimizing statistical learning algorithms to support longer-term learning over short-term gains requires sensitivity to the variability in the world.

The combined results of Experiments 1 and 2 provide initial evidence to support the hypothesis that children consider factors beyond the presence of uncertainty in making exploration decisions. In contrast to previous research (e.g., Levin et al., 2007), we find that preschoolers appropriately trade-off between exploration and exploitation as differences in expected value shift and the uncertainties before them become more or less advantageous. Furthermore, when faced with uncertainties about which they can form some expectation about possible outcomes, children’s exploration behavior was significantly different than when no information was available. They also appeared to show a bias towards exploring *beyond* expected value. It is possible that this reflects an additional motivation to reveal uncertainties, consistent with the characterization of young children as avid explorers, or a tendency towards ‘wishful thinking’ about the likelihood of getting a desirable outcome (see Wente et al., 2020). Experiment 3 provides insight into the possible motivations behind children’s decisions to explore by examining whether they are sensitive to the utility of information gained for future decision making.

## EXPERIMENT 3: UTILITY OF INFORMATION GAINED FOR FUTURE DECISION MAKING

The results thus far have shown that children are less inclined to explore uncertainties when they have no distributional information (Experiment 1) and use this information (when it is available) to guide their exploration decisions, with a slight tendency to explore over exploit (Experiment 2). Our third and final experiment asks whether children’s exploration decisions are sensitive to the potential for learning distributional information *from* their exploration. That is, sensitive to the potential utility of revealing uncertain outcomes, not from the reward amount, but from the *information gained* (Lindley, 1956; Shannon, 1948) to inform future explore-exploit decisions.

As in Experiments 1 and 2, each trial of the task in Experiment 3 offers participants a choice between two boxes: a known and an unknown that would be revealed if chosen. However, in this experiment, *both* options were drawn at random from a larger set of closed boxes. Before making their first choice, participants were shown the contents of several boxes in the known set and told they all contained the same reward amount. None of the boxes in the unknown set were revealed, and the distribution of reward amounts was initially unknown. Critically, therefore, a choice to explore the option drawn from this set also had the potential to improve children’s epistemic position for decisions on future trials by providing information about the initially ambiguous distribution of rewards in the unknown set.

In order to determine whether this potential utility of revealing new information influenced children’s decisions to explore, we compared choice behavior across three different conditions. In the *Feedback-Stay* condition, the contents of unknown boxes are revealed immediately following the participant’s choice to explore. In the *No-Feedback* condition, the contents of unknown boxes children chose to explore were not revealed until after all task trials were complete. In both conditions, exploring unknowns ultimately provided children with information about the unknown set. However, only in the *Feedback-Stay* condition could the resulting improvement in knowledge state be useful in making future decisions.

Of course, it is well-established that waiting on a reward decreases its subjective value (termed delay or temporal *discounting*) regardless of any other manipulation (e.g., Chung, 1965; Logan, 1965; see Liu et al., 2019 for this effect in 5-year-olds). Therefore, we also included a third condition to rule out the possibility that any differences between *No-Feedback* and *Feedback-Stay* were due merely to the difference in when unknowns were revealed. In the *Feedback-Switch* condition, the contents of selected unknown boxes were revealed immediately, but the two sets of boxes (both known and unknown) *changed* between each trial. Thus, although uncertainties could be immediately resolved through exploration, gaining information about the possible reward distribution in a set could not improve children’s knowledge of the environment for future decision-making.

If young children consider whether revealing uncertain outcomes can meaningfully improve their knowledge, then we would expect to see significantly more unknown box choices in *Feedback-Stay* than in the other two conditions. However, it is also possible that getting to resolve *any* uncertainty is appealing to young children, regardless of whether the information gained is consequential or supports learning. In this case, we would not expect a substantial difference in choice behavior between the *Feedback-Stay* and *Feedback-Switch* conditions, but we would expect less exploration in the *No-Feedback* condition where this reveal is delayed.

### Method.

#### Participants.

A total of 54 preschoolers (53.7% female, *M* = 57 months, *SD* = 5.41 months, Range = 46–68 months) were recruited from the same locations as Experiments 1 and 2 to participate in Experiment 3. Children were randomly assigned to one of three experimental conditions: *Feedback-Stay* (*n* = 18, *M* = 57.35 months, *SD* = 6.52 months), *No-Feedback* (*n* = 18, *M* = 57.31 months, *SD* = 4.68 months), and *Feedback-Switch* (*n* = 18, *M* = 57.1 months, *SD* = 5.18 months). Ten children were dropped and replaced either for failing to answer the practice question correctly (*n* = 9) or due to experimenter error (*n* = 1).

#### Materials.

A total of 192 new boxes were used in Experiment 3. As before, the visual appearance of the boxes indicated distinct ‘sets’ of identical boxes, but the number of boxes in a single set was increased to 16 to accommodate repeated draws. The same two sets of boxes (known and unknown) were used on all trials in both the *Feedback-Stay* and *No-Feedback* conditions. The *Feedback-Switch* condition used 12 sets of boxes (six known and six unknown) over the six task trials. All the boxes in known sets had two dots drawn inside, while boxes in the unknown sets contained between one and four dots in different proportions. Boxes in these sets were also surreptitiously marked to allow the experimenter to covertly control the amounts revealed when participants chose to explore. In the *Feedback-Switch* condition, the box sets were each arranged flat inside a clear zip-lock bag, then stacked inside two up-right paper bags. This allowed the experimenter to quickly switch the sets between trials while concealing the number of remaining trials. Three box sets were constructed and photographed to show participants during the introduction: the *large-set* had an average value of 15, the *medium-set* had an average value of 2, and the *small-set* had an average value of 0.6. See OSF at https://osf.io/cf87b/?view_only=555e65fef7c54093b53410e1d379a17b for materials used in each condition and recording of task procedure in the *Feedback-Stay* condition. The same practice boxes, marbles, and marble maze were also used.

#### Procedure.

**Figure 4.**
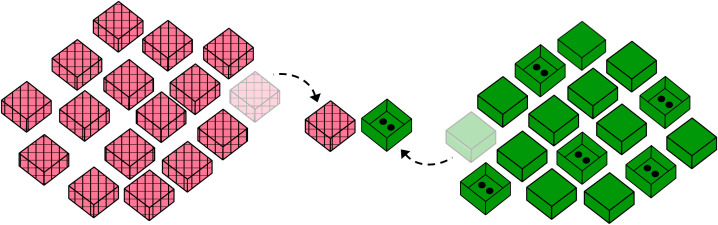
Setup of stimuli in a single task trial in Experiment 3.

The initial set-up of the task differed slightly between conditions: In the *No-Feedback* and *Feedback-Stay* conditions, two sets of boxes (one known and one unknown) were arranged on either side of the table out of reach of the participants and remained there for the entire duration of the task (Figure 4). In the *Feedback-Switch* condition, the sets were kept on the table inside of two upright paper bags (one bag of known sets and one of unknown). The experimenter brought out a new set of each kind and arranged them in the same way as in the other two conditions between each trial.

The introduction and practice were the same as in the previous experiments but followed by further instructions: The experimenter explained that there were different types of boxes and that different types contained different amounts of marbles. She used photographs of three box sets (distributions with expected values of 0.6, 2, and 15, respectively) to explain how boxes of the same type had the same external appearance and that types could have more or less marbles. Participants were then shown either the two different sets (in the *No-Feedback* and *Feedback-Stay* conditions) or the two different *groups* of sets (in the *Feedback-Switch* condition). For the known set(s), participants were told that all boxes contained two dots. For the unknown set(s), they were told that “we don’t know what the amounts of dots in any of the boxes are,” but that, like the sample pictures, boxes from the same set would have similar amounts inside.

The task consisted of six choice trials. On each trial, the experimenter would select one box (apparently at random) from each of the two sets. As in Experiments 1 and 2, the box from the known set was opened, the box from the unknown set remained closed, and participants were asked to choose between them. In the No-Feedback condition, all unknown boxes chosen were set aside and remained unopened until after the task trials. In contrast, unknown boxes selected in the *Feedback-Stay* and *Feedback-Switch* conditions were opened immediately after the participant’s choice. The experimenter revealed the amount inside the box but did not give participants the corresponding marbles until after the task trials.

In order to prevent the actual contents of unknown boxes from influencing decision-making behavior, the experimenter covertly tracked and controlled the outcomes revealed when children chose to explore. The first, third, and fifth times a child chose to explore always revealed two dots, and the second and fourth times revealed one and three dots (order counterbalanced across participants). To ensure that the average expected value of the unknown set(s) was roughly equivalent to the value of the known box, this was the order *regardless* of when and how often children chose the unknown box over the first five trials. For example, a child choosing to explore for the first time on the third trial would reveal a box containing two marbles rather than one or three). As it could not influence later choices, the unknown box on the sixth and final task trial always contained four dots regardless of how many previous exploration choices the child had made. The procedure for the *Feedback-Switch* condition was identical to *Feedback-Stay*, save that new sets of known and unknown boxes were used on each of the six task trials.

### Results and Discussion

**Figure 5.**
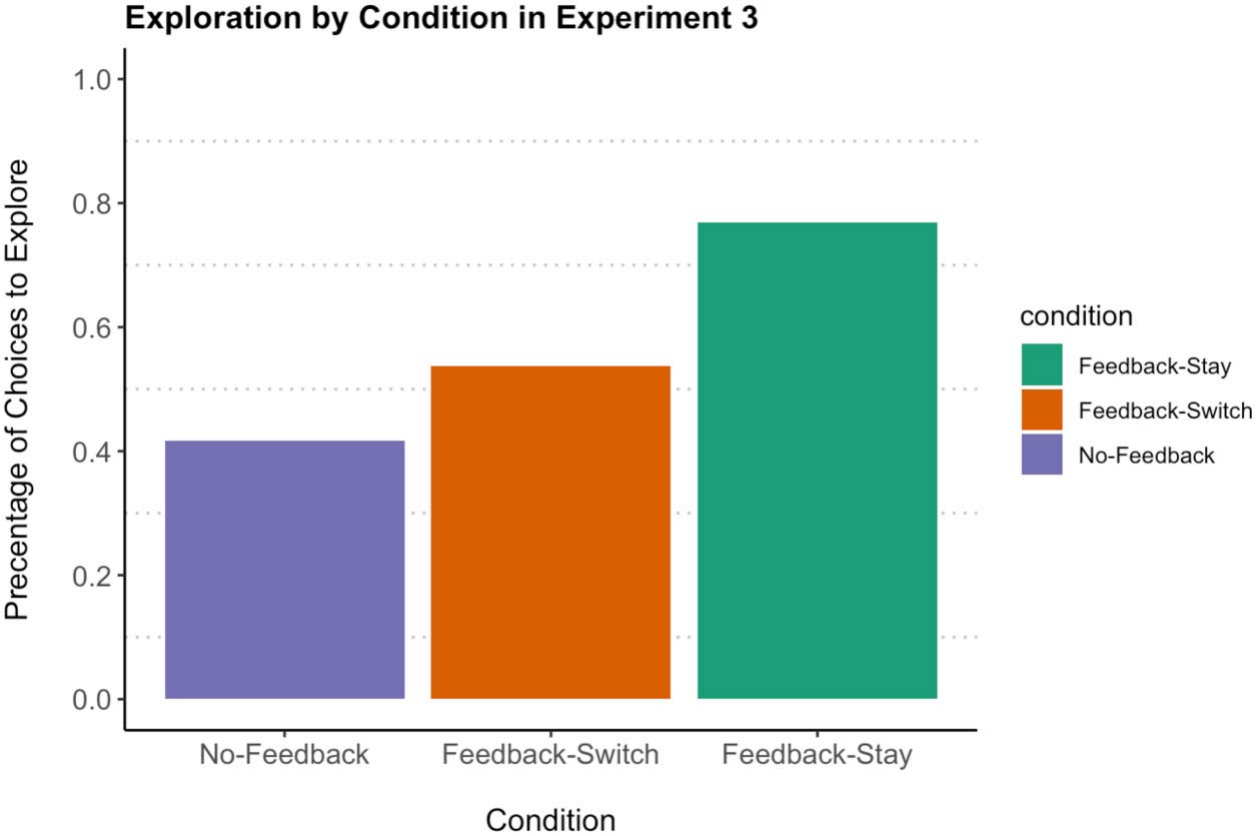
The proportion of exploration choices by condition in Experiment 3. *Note*. There were a total of 108 choice trials per condition. Error bars represent standard error.

Figure 5 shows the proportion of the 108 total trials in which children chose to explore in each condition in Experiment 3. We used a logistic mixed-effects model to predict choosing the unknown box based on condition, including random intercepts for participant and trial order. As in previous experiments, the model indicated substantial between-subjects variance (6.5) and no effect of trial order (0.2). A likelihood ratio test using the *lrtest* package revealed a significant overall effect of condition on choice to explore when this model was compared to a null model with random effects, X22(−2) = 15.51, *p* < 0.001.

Post-hoc pairwise comparisons, using the *emmeans* package with Tukey corrections for multiple comparisons, suggested that this effect was driven by children’s behavior in the *Feedback-Stay* condition. Exploration choices in the *Feedback-Stay* condition (76.85% of trials), were greater than in the *Feedback-Switch* condition (53.7% of trials, *p* = 0.04) or *No-Feedback* condition (41.67% of trials, *p* < 0.01). Participants explored significantly more often in the one condition where it was possible to learn about the unknown box set from the results of their exploration, even compared to the *Feedback-Switch* condition, which controlled for the effect of immediately revealing outcomes of uncertain options. Exploration choices in the two conditions in which it was not possible to learn from exploration, *No-Feedback* and *Feedback-Switch*, did not differ from each other (*p* = 0.4).

The differences between the conditions of Experiment 3 suggest that children’s decisions to explore were sensitive to whether or not exploration could meaningfully improve their knowledge. Looking within each condition: Participants in the *No-Feedback* condition chose the unknown box on only 41.67% of trials. While the intercept was not significantly different from chance (*p* = 0.21), this choice behavior suggests a reluctance to explore uncertain outcomes that is consistent with the results of Experiment 1 (choice behavior was also not significantly different between Experiment 1 and this condition, *p* = 0.19, two-tailed binomial). In the *Feedback-Switch* condition, children selected the unknown box on 53.7% of task trials, which was also not different from chance (*p* = 0.61). By contrast, children in the *Feedback-Stay* condition chose to explore on 76.85% of trials, which significantly differed from chance (*p* = 0.001).

In Experiment 3, children chose exploration significantly more often when revealing uncertainties was an opportunity to improve their knowledge of the overall task environment. This preference is especially striking since the *Feedback-Stay* condition presented children with an unknown box set that was initially *ambiguous*. The potential for learning may have encouraged children to overcome the reluctance to explore ambiguity seen in Experiment 1 and the *No-Feedback* condition. That said, it is not the case that subsequently reduced ambiguity on later trials of *Feedback-Stay* drove the difference between conditions: A comparison of choice behavior on the first task trial, when all participants had the same amount of information about the unknown set, showed the same difference between conditions (*p* < 0.05, *Pearson chi-square*). Another possible alternative explanation for this difference– that children were more willing to explore unknowns in *Feeedback-Stay* because of the lack of delay in carrying out their explorations– is ruled out by performance in the *Feedback-Switch* condition. The reward of immediately revealing uncertain outcomes via exploration, in the absence of any potential for learning from that exploration, led to children exploring only around half the time.

Critically, the actual outcomes revealed by exploration did *not* suggest to children that the unknown set would be more profitable in tangible rewards than the known set. The outcomes revealed in the *Feedback-Stay* condition were intentionally controlled so that the expected value of the unknown set remained roughly equivalent to the value of the known boxes. Nevertheless, there were marginally more choices to explore in this condition (76.85%) than in the 1:1 trials of Experiment 2 (60.42%), *t*(79) = 2, *p* = 0.05. Overall, the results of Experiment 3 suggest that, regardless of what tangible outcomes children expected or how quickly they were revealed, exploration of uncertainty was most appealing when it allowed children to improve their knowledge in a consequential way.

## GENERAL DISCUSSION

The current study investigates whether preschoolers’ exploration decisions are sensitive to factors beyond the presence and opportunity for resolving uncertainty. In three experiments, we tested whether ambiguity, expected value, and potential to inform future decision-making influenced choice behavior in an explore-exploit task. While previous research provides ample evidence for the influence of these three contextual factors on adult decision-making, it was unclear whether preschoolers employ similar mechanisms in choosing when to explore.

In Experiment 1, most 4- to 5-year-olds chose *not* to explore in ambiguous circumstances. While not the classic ambiguity aversion effect typically examined in adults, the behavior was a striking contrast to young children’s well-documented tendency to explore uncertain or novel outcomes. Instead, these results demonstrate that children choose to forego opportunities to explore when factors beyond the uncertainty make it less appealing. This finding suggests that young children are sensitive to the different *types* of uncertainty that can arise in unfamiliar environments. Preschoolers weigh ambiguous uncertainties differently in their decision-making than uncertainties for which they have distribution information, suggesting that our early developing preference for approaching and revealing unknowns in our surroundings is not an indiscriminate impulse.

In Experiment 2, we modified our task to include information about the possible outcomes of uncertain options. By providing the distribution of reward amounts in each box set, we explored whether children consider expected value when deciding whether to explore or exploit. Performance on this task revealed two main findings. First, children chose exploration significantly more often than in Experiment 1. The change from avoiding exploration under ambiguity observed in Experiment 1 suggests that children were sensitive to the presence of distributional information in their explore-exploit decisions. Second, children also made use of this information, considering the expected values of the sets from which unknown boxes were drawn, to guide their decisions. As the expected value in the sets increased, so did children’s choices to explore the unknown box over exploiting the known one. Importantly, because we controlled for lower values in the distributional set, children could not use a simple heuristic to guide decisions (e.g., choosing boxes from sets with only high outcome values). In contrast to previous developmental research, we found that children as young as four inferred the expected value of uncertain outcomes and accurately drew from these expectations when considering whether to explore uncertainties in their environment.

In Experiment 3, we finally observed the strong preference for exploring uncertain outcomes considered a hallmark of early childhood (Gopnik, 2020). However, even in this case, children only showed this preference when revealing uncertainties was an opportunity to gain information about an initially ambiguous aspect of the task environment. Comparison of choice behavior across conditions and experiments rules out the possibility that children in the *Feedback-Stay* condition were motivated either by immediately revealing unknowns or the expected value of their contents. Instead, our results suggest that exploration is coherently motivated, even in young children, by consideration of the potential utility of revealing uncertain information. These results are consistent with theoretical accounts of the role of learning in driving exploratory bias in early childhood and demonstrate a surprisingly sophisticated sensitivity to whether information gained by exploration will support later decisions.

### Limitations and Future Work

We were fortunate to be able to recruit from a socio-economically and racially diverse population of preschoolers for these experiments, providing some suggestive evidence that our results may generalize across other populations. However, our sample was restricted to children in the Eastern United States. While there is no theoretical motivation to expect that the general trends found here would differ across a more representative global population, it will be important for future work to replicate these findings in additional populations. For example, there is good reason to believe that children who have experienced early adversity may have different explore-exploit thresholds. While we might predict that all children would similarly be sensitive to differences in expected values and utilities of revealing information for future decision-making, it is possible that preferences for exploitation (Humphreys et al., 2015; Lloyd et al., 2022) and greater discounting of future reward (Frankenhuis et al., 2016) would lead to different choice behavior in these populations. Future work must probe more deeply into the ways in which individual and community-level life experiences might contribute to how children negotiate the explore-exploit trade-offs examined here.

The choice to focus the current investigation on preschool-aged children was motivated by apparently contradictory findings of prior work on explore-exploit decisions in this age group. Our goal was to support this growing literature by clarifying the roles of sensitivity to uncertainty, ambiguity, expected value, and potential to inform future decisions in driving exploration. This is also a critical age, given that children are just about to enter formal schooling. However, there is no reason to believe that sensitivity to these factors begins at 4- or 5-years-of-age. Indeed, a growing body of research has pointed to early core meta-cognitive abilities that reflect sensitivity to uncertainty and information monitoring in the first two years of life (Begus & Southgate, 2018; Goupil & Kouider, 2019; Goupil et al., 2016; Kidd & Hayden, 2015). As new tools are developed for infant work, such as coupling EEG, eye-tracking, and behavioral methods (see Begus et al., 2015; Begus & Bonawitz, 2020), it may be possible to extend these questions to younger populations and better understand whether and how these sensitivities develop from infancy to early childhood.

Finally, the current work does not intend to be in any way a complete characterization of factors influencing early exploration decisions. For example, the results of Experiment 2 suggested a potentially linear relationship between expected value and exploration. However, it is challenging to precisely model or draw strong conclusions about the exact nature of this relationship without larger samples of observations. Furthermore, while the differences between conditions in Experiment 3 bears a similarity to the influence of time-horizons in adult explore-exploit behavior, the design was not intended to determine the specific influence of time-horizons on children’s exploration decisions. Examining these factors is outside the scope of the current research questions and would require engaging children in new manipulations and over greater numbers of trials. Future work may develop and explore these questions to help determine a more precise account of how young children weigh exploration decisions.

### Connections to Curiosity and Belief-Revision

Understanding when and why children are motivated to learn has been a long-standing concern for education. Indeed, Piaget’s view was, “The principle goal of education is to create [people] who are … creative, inventive, and discoverers” (Duckworth, 1964, p.175). Curiosity has also re-emerged in recent years as a core area of interest in psychology, neuroscience, and machine learning. Efforts to define curiosity’s role in intelligence and learning at behavioral and neural levels underline the importance of understanding the factors guiding exploratory decision-making during development. However, studying curiosity comes with the challenge of trying to learn about the ‘itch’ of an internal epistemic state from measuring the ‘scratch’ of external behavior. Using behavioral exploration as the solitary metric for characterizing curiosity risks missing the myriad additional factors that may relate to exploration decisions. Agents of all ages might choose to explore for reasons unrelated to curiosity, such as a lure of profit beyond knowledge. Conversely, a learner may choose not to explore despite their curiosity because they feel it is not permitted, will not be rewarded, or if other costs are too high. The influence of these additional utilities may change with age and experience, further muddying how we interpret exploration behavior and making it challenging to characterize shifts in curiosity across the life-span. This makes it particularly critical, therefore, to improve our understanding of the decision-making trade-offs in very young learners’ exploration. We see essential links between the growing broader research into curiosity and our current work to identify and chart the factors that motivate young learners to scratch this itch.

There are also intriguing connections between the current work and broader questions about learning as belief-revision. Learning requires engaging in a particular form of explore-exploit trade-off: whether to continue exploiting a belief we currently hold or to conduct a mental search for a potentially better alternative. The decision to ‘mentally’ explore may depend on much the same mechanisms for evaluating reward and cost (estimations of utility, expected value, etc.) as our exploration of the external world. For example, learners might weigh the reward of exploiting a current belief in terms of maintaining explanatory coherence against the cost of dissatisfaction with the accuracy of the belief’s predictions. In the case of evaluating exploration, the potential for increasing explanatory coherence may be weighed against the costs of the cognitive effort required to ‘search’ mental space for new ideas. Just as the preschoolers in our study weighed the potential of exploration to inform future decision-making, a learner might consider whether the effort expended on mental search outweighs the benefit to future explanatory reasoning. Such behavior would be consistent with computational models of belief revision (e.g., Bonawitz, Denison, Gopnik, & Griffiths, 2014), resource-rational decision-making (e.g., Lieder & Griffiths, 2020, see Persaud et al., 2020 for developmental commentary), and recent empirical evidence that preschoolers trade-off cognitive effort and expected reward in deciding whether to tackle new problems (Wang & Bonawitz, 2022). Our finding that preschoolers’ explore-exploit decisions are sensitive to the expected value and utility of information gain offers novel avenues for better understanding choices to adhere to or abandon beliefs during learning.

### Summary

Taken together, the evidence from these three experiments suggests that, despite children’s well-known preference to explore the unknown and unfamiliar in their surroundings, they do not do so indiscriminately. Instead, children’s decision to explore involves consideration of numerous factors beyond the uncertainty of the outcome itself. We find that preschoolers’ exploration behavior is sensitive to a number of complex and contextual factors, including: whether they have any expectation about what the to-be-explored environment is actually like (Experiment 1), what the possible outcomes of an exploration might be (Experiment 2), and whether or not exploration will reveal information that can consequently improve their current knowledge state (Experiment 3). These findings add to the growing support for the claim that there is a careful and considered decision-making process guiding exploratory behavior in early childhood. It also goes beyond previous work to show how decision-making in explore-exploit contexts is influenced by sensitivity factors previously demonstrated in adults. Expanding our understanding of early exploration beyond the mere presence of uncertainty allows us to explain not only why exploration is so ubiquitous in early childhood, but also why it is so essential and rewarding: it supports learning.

## ACKNOWLEDGMENTS

We would like to thank Luke Miratrix and Junyi Chu for statistical consultation, the schools and children who participated in the project, and members of the CoCoDev lab for feedback on earlier versions of this work. We would also like to acknowledge the construction crew at Rutgers University, Newark that drilled a hole into our exterior lab wall accidentally and then, in an attempt to repair it, dumped several pounds of concrete on the stimuli used in this study.

## FUNDING INFORMATION

This work was supported in part by the James S. McDonnell Foundation (JSMF) and Jacobs Foundation (EB).

## DATA AVAILABILITY STATEMENT

The data that support the findings of this study are openly available on Open Science Framework at https://doi.org/10.17605/OSF.IO/CF87B.

## AUTHOR CONTRIBUTIONS

Elizabeth Lapidow: Conceptualization, Methodology, Formal Analysis, Investigation, Writing – Original Draft, Visualization. Elizabeth Bonawitz: Conceptualization, Methodology, Formal Analysis, Resources, Writing – Review & Editing, Supervision, Project Administration, Funding Acquisition.

## Note

^1^ In order to establish the success of this procedure in preventing children from tracking desirable boxes and choosing to explore based on whether or not these were selected, we compared the amount of marbles children won from unknown boxes to the average value of box sets. We find from this analysis that the difference (*M* = −0.05, *SD* = 1.97) was not statistically different from zero, *t*(92) = −0.26, *p* = 0.79, ruling out the concern that children’s choices were biased by tracking the sampled boxes.

## References

[bib1] Anderson, N. H. (1980). Information integration theory in developmental psychology. In F. Wilkening, J. Becker, & T. Trabasso (Eds.), Information integration by children (pp. 1–45). Erlbaum.

[bib2] Baldwin, D. A., Markman, E. M., & Melartin, R. L. (1993). Infants’ ability to draw inferences about nonobvious object properties: Evidence from exploratory play. Child Development, 64(3), 711–728. 10.2307/1131213, 8339691

[bib3] Bayless, S., & Schlottmann, A. (2010). Skill-related uncertainty and expected value in 5- to 7-year-olds. Psicologica, 31(3), 677–687.

[bib4] Becker, S. W., & Brownson, F. O. (1964). What price ambiguity? Or the role of ambiguity in decision-making. Journal of Political Economy, 72, 62–73. 10.1086/258854

[bib5] Begus, K., & Bonawitz, E. (2020). The rhythm of learning: Theta oscillations as an index of active learning in infancy. Developmental Cognitive Neuroscience, 45, Article 100810. 10.1016/j.dcn.2020.100810, 33040970 PMC7371744

[bib6] Begus, K., & Southgate, V. (2018). Curious learners: How infants’ motivation to learn shapes and is shaped by infants’ interactions with the social world. In M. M. Saylor & P. A. Ganea (Eds.), Active learning from infancy to childhood: Social motivation, cognition, and linguistic mechanisms (pp. 13–37). Springer. 10.1007/978-3-319-77182-3_2

[bib7] Begus, K., Southgate, V., & Gliga, T. (2015). Neural mechanisms of infant learning: Differences in frontal theta activity during object exploration modulate subsequent object recognition. Biology Letters, 11(5), Article 20150041. 10.1098/rsbl.2015.0041, 26018832 PMC4455734

[bib8] Betsch, T., & Lang, A. (2013). Utilization of probabilistic cues in the presence of irrelevant information: A comparison of risky choice in children and adults. Journal of Experimental Child Psychology, 115(1), 108–125. 10.1016/j.jecp.2012.11.003, 23403227

[bib9] Blanco, N. J., & Sloutsky, V. M. (2021). Systematic exploration and uncertainty dominate young children’s choices. Developmental Science, 24(2), Article e13026. 10.1111/desc.13026, 32767496 PMC7867663

[bib10] Blankenstein, N. E., Crone, E. A., van den Bos, W., & van Duijvenvoorde, A. C. K. (2016). Dealing with uncertainty: Testing risk- and ambiguity-attitude across adolescence. Developmental Neuropsychology, 41(1–2), 77–92. 10.1080/87565641.2016.1158265, 27028162

[bib11] Bonawitz, E., Denison, S., Gopnik, A., & Griffiths, T. L. (2014). Win-Stay, Lose-Sample: A simple sequential algorithm for approximating Bayesian inference. Cognitive Psychology, 74, 35–65. 10.1016/j.cogpsych.2014.06.003, 25086501

[bib12] Bonawitz, E., Denison, S., Griffiths, T. L., & Gopnik, A. (2014). Probabilistic models, learning algorithms, and response variability: Sampling in cognitive development. Trends in Cognitive Sciences, 18(10), 497–500. 10.1016/j.tics.2014.06.006, 25001609

[bib13] Bonawitz, E. B., van Schijndel, T. J. P., Friel, D., & Schulz, L. E. (2012). Children balance theories and evidence in exploration, explanation, and learning. Cognitive Psychology, 64(4), 215–234. 10.1016/j.cogpsych.2011.12.002, 22365179

[bib14] Bruner, J. S., Jolly, A., & Sylva, K. (1976). Play: Its role in development and evolution. Penguin.

[bib15] Camerer, C., & Weber, M. (1992). Recent developments in modeling preferences: Uncertainty and ambiguity. Journal of Risk and Uncertainty, 5(4), 325–370. 10.1007/BF00122575

[bib16] Chung, S.-H. (1965). Effects of delayed reinforcement in a concurrent situation. Journal of the Experimental Analysis of Behavior, 8(6), 439–444. 10.1901/jeab.1965.8-439, 5851405 PMC1338130

[bib18] Cook, C., Goodman, N. D., & Schulz, L. E. (2011). Where science starts: Spontaneous experiments in preschoolers’ exploratory play. Cognition, 120(3), 341–349. 10.1016/j.cognition.2011.03.003, 21561605

[bib19] Davidson, D. (1991). Developmental differences in children’s search of predecisional information. Journal of Experimental Child Psychology, 52(2), 239–255. 10.1016/0022-0965(91)90061-V

[bib20] Denison, S., Bonawitz, E., Gopnik, A., & Griffiths, T. L. (2013). Rational variability in children’s causal inferences: The sampling hypothesis. Cognition, 126(2), 285–300. 10.1016/j.cognition.2012.10.010, 23200511

[bib21] Denison, S., Konopczynski, K., Garcia, V., & Xu, F. (2006). Probabilistic reasoning in preschoolers: Random sampling and base rate. In R. Sun & N. Miyake (Eds.), Proceedings of the 28th Annual Conference of the Cognitive Science Society (pp. 1216–1221). Psychology Press.

[bib22] Denison, S., & Xu, F. (2010). Twelve- to 14-month-old infants can predict single-event probability with large set sizes. Developmental Science, 13(5), 798–803. 10.1111/j.1467-7687.2009.00943.x, 20712746

[bib23] Denison, S., & Xu, F. (2014). The origins of probabilistic inference in human infants. Cognition, 130(3), 335–347. 10.1016/j.cognition.2013.12.001, 24384147

[bib24] Duckworth, E. (1964). Piaget rediscovered. The Arithmetic Teacher, 11(7), 496–499. 10.5951/AT.11.7.0496

[bib25] Ellsberg, D. (1961). Risk, ambiguity, and the savage axioms. The Quarterly Journal of Economics, 75(4), 643–669. 10.2307/1884324

[bib26] Feather, N. T. (1982). Expectations and actions: Expectancy-value models in psychology. Erlbaum.

[bib27] Frank, M. J., Doll, B. B., Oas-Terpstra, J., & Moreno, F. (2009). Prefrontal and striatal dopaminergic genes predict individual differences in exploration and exploitation. Nature Neuroscience, 12(8), 1062–1068. 10.1038/nn.2342, 19620978 PMC3062477

[bib28] Frankenhuis, W. E., Panchanathan, K., & Nettle, D. (2016). Cognition in harsh and unpredictable environments. Current Opinion in Psychology, 7, 76–80. 10.1016/j.copsyc.2015.08.011

[bib29] Frisch, D., & Baron, J. (1988). Ambiguity and rationality. Journal of Behavioral Decision Making, 1(3), 149–157. 10.1002/bdm.3960010303

[bib30] Fu, W.-T., & Gray, W. D. (2006). Suboptimal tradeoffs in information seeking. Cognitive Psychology, 52(3), 195–242. 10.1016/j.cogpsych.2005.08.002, 16356487

[bib31] Garon, N., & Moore, C. (2004). Complex decision-making in early childhood. Brain and Cognition, 55(1), 158–170. 10.1016/S0278-2626(03)00272-0, 15134850

[bib32] Gigerenzer, G., Dieckmann, A., & Gaissmaier, W. (2012). Efficient cognition through limited search. In Ecological rationality: Intelligence in the world (pp. 241–273). Oxford University Press. 10.1093/acprof:oso/9780195315448.003.0075

[bib33] Gittins, J. C., & Jones, D. M. (1974). A dynamic allocation index for the sequential design of experiments. In J. Gani, K. Sarkadi, & I. Vineze (Eds.), Progress in statistics (pp. 241–266). North Holland.

[bib34] Golinkoff, R. M., Hirsh-Pasek, K., & Singer, D. G. (2006). Why play = learning: A challenge for parents and educators. In D. G. Singer, R. M. Golinkoff, & K. Hirsh-Pasek (Eds.), Play = learning: How play motivates and enhances children’s cognitive and social-emotional growth (pp. 3–12). Oxford University Press. 10.1093/acprof:oso/9780195304381.003.0001

[bib35] Gopnik, A. (2020). Childhood as a solution to explore–exploit tensions. Philosophical Transactions of the Royal Society B, 375(1803), Article 20190502. 10.1098/rstb.2019.0502, 32475327 PMC7293160

[bib36] Gopnik, A., & Wellman, H. M. (2012). Reconstructing constructivism: Causal models, Bayesian learning mechanisms, and the theory theory. Psychological Bulletin, 138(6), 1085–1108. 10.1037/a0028044, 22582739 PMC3422420

[bib37] Goupil, L., & Kouider, S. (2019). Developing a reflective mind: From core metacognition to explicit self-reflection. Current Directions in Psychological Science, 28(4), 403–408. 10.1177/0963721419848672

[bib38] Goupil, L., Romand-Monnier, M., & Kouider, S. (2016). Infants ask for help when they know they don’t know. Proceedings of the National Academy of Sciences of the United States of America, 113(13), 3492–3496. 10.1073/pnas.1515129113, 26951655 PMC4822620

[bib39] Gweon, H., & Schulz, L. E. (2008). Stretching to learn: Ambiguous evidence and variability in preschoolers exploratory play. In B. C. Love, K. McRae, & V. M. Sloutsky (Eds.), Proceedings of the 30th Annual Conference of the Cognitive Science Society (pp. 570–574). Cognitive Science Society.

[bib40] Gweon, H., Tenenbaum, J. B., & Schulz, L. E. (2010). Infants consider both the sample and the sampling process in inductive generalization. Proceedings of the National Academy of Sciences of the United States of America, 107(20), 9066–9071. 10.1073/pnas.1003095107, 20435914 PMC2889113

[bib41] Heath, C., & Tversky, A. (1991). Preference and belief: Ambiguity and competence in choice under uncertainty. Journal of Risk and Uncertainty, 4(1), 5–28. 10.1007/BF00057884

[bib42] Humphreys, K. L., Lee, S. S., Telzer, E. H., Gabard-Durnam, L. J., Goff, B., Flannery, J., & Tottenham, N. (2015). Exploration–exploitation strategy is dependent on early experience. Developmental Psychobiology, 57(3), 313–321. 10.1002/dev.21293, 25783033 PMC5934758

[bib43] Huizenga, H. M., Crone, E. A., & Jansen, B. J. (2007). Decision-making in healthy children, adolescents and adults explained by the use of increasingly complex proportional reasoning rules. Developmental Science, 10(6), 814–825. 10.1111/j.1467-7687.2007.00621.x, 17973798

[bib44] Juni, M. Z., Gureckis, T. M., & Maloney, L. T. (2016). Information sampling behavior with explicit sampling costs. Decision, 3(3), 147–168. 10.1037/dec0000045, 27429991 PMC4942190

[bib45] Kidd, C., & Hayden, B. Y. (2015). The psychology and neuroscience of curiosity. Neuron, 88(3), 449–460. 10.1016/j.neuron.2015.09.010, 26539887 PMC4635443

[bib46] Kidd, C., Piantadosi, S. T., & Aslin, R. N. (2012). The Goldilocks effect: Human infants allocate attention to visual sequences that are neither too simple nor too complex. PLoS ONE, 7(5), Article e36399. 10.1371/journal.pone.0036399, 22649492 PMC3359326

[bib47] Kidd, C., Piantadosi, S. T., & Aslin, R. N. (2014). The Goldilocks effect in infant auditory attention. Child Development, 85(5), 1795–1804. 10.1111/cdev.12263, 24990627 PMC4807134

[bib48] Knox, W. B., Otto, A. R., Stone, P., & Love, B. C. (2012). The nature of belief-directed exploratory choice in human decision-making. Frontiers in Psychology, 2, Article 398. 10.3389/fpsyg.2011.00398, 22319503 PMC3269072

[bib49] Kushnir, T., & Gopnik, A. (2005). Young children infer causal strength from probabilities and interventions. Psychological Science, 16(9), 678–683. 10.1111/j.1467-9280.2005.01595.x, 16137252

[bib50] Lapidow, E., Goddu, M. K., & Walker, C. M. (2022). Reasoning from samples to populations: Children use variability information to predict novel outcomes. In J. Culbertson, A. Perfors, H. Rabagliati, & V. Ramenzoni (Eds.), Proceedings of the 44th Annual Conference of the Cognitive Science Society (pp. 2193–2199). Cognitive Science Society.

[bib51] Lapidow, E., Killeen, I., & Walker, C. M. (2022). Learning to recognize uncertainty vs. recognizing uncertainty to learn: Confidence judgments and exploration decisions in preschoolers. Developmental Science, 25(2), Article e13178. 10.1111/desc.13178, 34596300

[bib52] Lapidow, E., Tandon, T., Goddu, M., & Walker, C. M. (2021). A tale of three platforms: Investigating preschoolers’ second-order inferences using in-person, Zoom, and Lookit methodologies. Frontiers in Psychology, 12, Article 731404. 10.3389/fpsyg.2021.731404, 34721195 PMC8548456

[bib53] Lauriola, M., & Levin, I. P. (2001). Relating individual differences in attitude toward ambiguity to risky choices. Journal of Behavioral Decision Making, 14(2), 107–122. 10.1002/bdm.368

[bib54] Lee, M. D., Zhang, S., Munro, M., & Steyvers, M. (2011). Psychological models of human and optimal performance in bandit problems. Cognitive Systems Research, 12(2), 164–174. 10.1016/j.cogsys.2010.07.007

[bib55] Levin, I. P., Weller, J. A., Pederson, A., & Harshman, L. (2007). Age-related differences in adaptive decision making: Sensitivity to expected value in risky choice. Judgment and Decision Making, 2(4), 225–233. 10.1017/S1930297500000553

[bib56] Li, R., Brannon, E. M., & Huettel, S. A. (2015). Children do not exhibit ambiguity aversion despite intact familiarity bias. Frontiers in Psychology, 5, Article 1519. 10.3389/fpsyg.2014.01519, 25601848 PMC4283450

[bib57] Li, R., Roberts, R. C., Huettel, S. A., & Brannon, E. M. (2017). Five-year-olds do not show ambiguity aversion in a risk and ambiguity task with physical objects. Journal of Experimental Child Psychology, 159, 319–326. 10.1016/j.jecp.2017.02.013, 28359540

[bib58] Lieder, F., & Griffiths, T. L. (2020). Resource-rational analysis: Understanding human cognition as the optimal use of limited computational resources. Behavioral and Brain Sciences, 43, Article E1. 10.1017/S0140525X1900061X, 30714890

[bib59] Lindley, D. V. (1956). On a measure of the information provided by an experiment. The Annals of Mathematical Statistics, 27(4), 986–1005. 10.1214/aoms/1177728069

[bib60] Liquin, E. G., Callaway, F., & Lombrozo, T. (2021). Developmental change in what elicits curiosity. In Proceedings of the 43rd Annual Conference of the Cognitive Science Society (pp. 1360–1366). Cognitive Science Society.

[bib209] Liquin, E. G., & Lombrozo, T. (2020). Explanation-seeking curiosity in childhood. Current Opinion in Behavioral Sciences, 35, 14–20. 10.1016/j.cobeha.2020.05.012

[bib61] Liu, S., Gonzalez, G., & Warneken, F. (2019). Worth the wait: Children trade off delay and reward in self- and other-benefiting decisions. Developmental Science, 22(1), Article e12702. 10.1111/desc.12702, 29978941

[bib62] Lloyd, A., McKay, R. T., & Furl, N. (2022). Individuals with adverse childhood experiences explore less and underweight reward feedback. Proceedings of the National Academy of Sciences of the United States of America, 119(4), Article e2109373119. 10.1073/pnas.2109373119, 35046026 PMC8794829

[bib63] Logan, F. A. (1965). Decision making by rats: Delay versus amount of reward. Journal of Comparative and Physiological Psychology, 59, 1–12. 10.1037/h0021633, 14282403

[bib64] Meder, B., Wu, C. M., Schulz, E., & Ruggeri, A. (2021). Development of directed and random exploration in children. Developmental Science, 24(4), Article e13095. 10.1111/desc.13095, 33539647

[bib65] Mehlhorn, K., Newell, B. R., Todd, P. M., Lee, M. D., Morgan, K., Braithwaite, V. A., Hausmann, D., Fiedler, K., & Gonzalez, C. (2015). Unpacking the exploration–exploitation tradeoff: A synthesis of human and animal literatures. Decision, 2(3), 191–215. 10.1037/dec0000033

[bib66] Meier, K. M., & Blair, M. R. (2013). Waiting and weighting: Information sampling is a balance between efficiency and error-reduction. Cognition, 126(2), 319–325. 10.1016/j.cognition.2012.09.014, 23099124

[bib67] Meyer, R. J., & Shi, Y. (1995). Sequential choice under ambiguity: Intuitive solutions to the armed-bandit problem. Management Science, 41(5), 817–834. 10.1287/mnsc.41.5.817

[bib68] Newell, B. R., & Shanks, D. R. (2003). Take the best or look at the rest? Factors influencing “one-reason” decision making. Journal of Experimental Psychology: Learning, Memory, and Cognition, 29(1), 53–65. 10.1037/0278-7393.29.1.53, 12549583

[bib69] Nussenbaum, K., Cohen, A. O., Davis, Z. J., Halpern, D. J., Gureckis, T. M., & Hartley, C. A. (2020). Causal information-seeking strategies change across childhood and adolescence. Cognitive Science, 44(9), Article e12888. 10.1111/cogs.12888, 32882077

[bib70] Persaud, K., Bass, I., Colantonio, J., Macias, C., & Bonawitz, E. (2020). Opportunities and challenges integrating resource-rational analysis with developmental perspectives. Behavioral and Brain Sciences, 43, Article E18. 10.1017/S0140525X19001560, 32159498

[bib71] Piaget, J. (1930). The child’s conception of physical causality. Harcourt, Brace.

[bib72] Plate, R. C., Fulvio, J. M., Shutts, K., Green, C. S., & Pollak, S. D. (2018). Probability learning: Changes in behavior across time and development. Child Development, 89(1), 205–218. 10.1111/cdev.12718, 28121026 PMC5526727

[bib73] Rich, A. S., & Gureckis, T. M. (2014). The value of approaching bad things. In Proceedings of the 36th Annual Conference of the Cognitive Science Society (pp. 1281–1286). Cognitive Science Society.

[bib74] Rich, A. S., & Gureckis, T. M. (2018). Exploratory choice reflects the future value of information. Decision, 5(3), 177–192. 10.1037/dec0000074

[bib76] Ruggeri, A., Lombrozo, T., Griffiths, T. L., & Xu, F. (2016). Sources of developmental change in the efficiency of information search. Developmental Psychology, 52(12), 2159–2173. 10.1037/dev0000240, 27893251

[bib77] Schlottmann, A. (2000). Children’s judgements of gambles: A disordinal violation of utility. Journal of Behavioral Decision Making, 13(1), 77–89. 10.1002/(SICI)1099-0771(200001/03)13:1<77::AID-BDM344>3.0.CO;2-Y

[bib78] Schlottmann, A. (2001). Children’s probability intuitions: Understanding the expected value of complex gambles. Child Development, 72(1), 103–122. 10.1111/1467-8624.00268, 11280473

[bib79] Schlottmann, A., & Anderson, N. H. (1994). Children’s judgments of expected value. Developmental Psychology, 30(1), 56–66. 10.1037/0012-1649.30.1.56

[bib80] Schlottmann, A., & Tring, J. (2005). How children reason about gains and losses: Framing effects in judgement and choice. Swiss Journal of Psychology, 64(3), 153–171. 10.1024/1421-0185.64.3.153

[bib81] Schulz, E., Wu, C. M., Ruggeri, A., & Meder, B. (2019). Searching for rewards like a child means less generalization and more directed exploration. Psychological Science, 30(11), 1561–1572. 10.1177/0956797619863663, 31652093

[bib82] Schulz, L. E. (2012). The origins of inquiry: Inductive inference and exploration in early childhood. Trends in Cognitive Sciences, 16(7), 382–389. 10.1016/j.tics.2012.06.004, 22721579

[bib83] Schulz, L. E., & Bonawitz, E. B. (2007). Serious fun: Preschoolers engage in more exploratory play when evidence is confounded. Developmental Psychology, 43(4), 1045–1050. 10.1037/0012-1649.43.4.1045, 17605535

[bib84] Schulz, L. E., Standing, H. R., & Bonawitz, E. B. (2008). Word, thought, and deed: The role of object categories in children’s inductive inferences and exploratory play. Developmental Psychology, 44(5), 1266–1276. 10.1037/0012-1649.44.5.1266, 18793061

[bib85] Shannon, C. E. (1948). A mathematical theory of communication. Bell System Technical Journal, 27(3), 379–423. 10.1002/j.1538-7305.1948.tb01338.x

[bib86] Shanteau, J. (1975). An information integration analysis of risky decision making. In M. F. Kaplan & S. Schwartz (Eds.), Human judgment and decision processes (pp. 109–137). Academic Press. 10.1016/B978-0-12-397250-7.50011-8

[bib87] Siegel, M. H., Magid, R. W., Pelz, M., Tenenbaum, J. B., & Schulz, L. E. (2021). Children’s exploratory play tracks the discriminability of hypotheses. Nature Communications, 12(1), Article 3598. 10.1038/s41467-021-23431-2, 34127657 PMC8203670

[bib88] Somerville, L. H., Sasse, S. F., Garrad, M. C., Drysdale, A. T., Abi Akar, N., Insel, C., & Wilson, R. C. (2017). Charting the expansion of strategic exploratory behavior during adolescence. Journal of Experimental Psychology: General, 146(2), 155–164. 10.1037/xge0000250, 27977227

[bib89] Stahl, A. E., & Feigenson, L. (2015). Observing the unexpected enhances infants’ learning and exploration. Science, 348(6230), 91–94. 10.1126/science.aaa3799, 25838378 PMC5861377

[bib91] Teodorescu, K., & Erev, I. (2014). On the decision to explore new alternatives: The coexistence of under- and over-exploration. Journal of Behavioral Decision Making, 27(2), 109–123. 10.1002/bdm.1785

[bib92] Trautmann, S. T., & van de Kuilen, G. (2015). Ambiguity attitudes. In G. Keren & G. Wu (Eds.), The Wiley-Blackwell handbook of judgment and decision making (pp. 89–116). John Wiley & Sons, Inc. 10.1002/9781118468333.ch3

[bib93] van Schijndel, T. J. P., Visser, I., van Bers, B. M. C. W., & Raijmakers, M. E. J. (2015). Preschoolers perform more informative experiments after observing theory-violating evidence. Journal of Experimental Child Psychology, 131, 104–119. 10.1016/j.jecp.2014.11.008, 25544394

[bib94] Wang, J. J., & Bonawitz, E. (2022). Children integrate task difficulty and reward probability when deciding when to give up. Journal of Cognition and Development, 1–13.37614812

[bib95] Wang, J., Yang, Y., Macias, C., & Bonawitz, E. (2021). Children with more uncertainty in their intuitive theories seek domain-relevant information. Psychological Science, 32(7), 1147–1156. 10.1177/0956797621994230, 34180722 PMC8641137

[bib96] Weisler, A., & McCall, R. R. (1976). Exploration and play: Resume and redirection. American Psychologist, 31(7), 492–508. 10.1037/0003-066X.31.7.492

[bib97] Wente, A. O., Goddu, M. K., Garcia, T., Posner, E., Fernández Flecha, M., & Gopnik, A. (2020). Young children are wishful thinkers: The development of wishful thinking in 3- to 10-year-old children. Child Development, 91(4), 1166–1182. 10.1111/cdev.13299, 31400006

[bib98] Wilson, R. C., Bonawitz, E., Costa, V. D., & Ebitz, R. B. (2021). Balancing exploration and exploitation with information and randomization. Current Opinion in Behavioral Sciences, 38, 49–56. 10.1016/j.cobeha.2020.10.001, 33184605 PMC7654823

[bib99] Wilson, R. C., Geana, A., White, J. M., Ludvig, E. A., & Cohen, J. D. (2014). Humans use directed and random exploration to solve the explore-exploit dilemma. Journal of Experimental Psychology: General, 143(6), 2074–2081. 10.1037/a0038199, 25347535 PMC5635655

[bib100] Wright, G. N. (1984). Behavioral decision theory: An introduction. Sage Publications.

[bib101] Xu, F., & Garcia, V. (2008). Intuitive statistics by 8-month-old infants. Proceedings of the National Academy of Sciences of the United States of America, 105(13), 5012–5015. 10.1073/pnas.0704450105, 18378901 PMC2278207

[bib102] Zhuang, W., Niebaum, J., & Munakata, Y. (2023). Changes in adaptation to time horizons across development. Developmental Psychology, 59(8), 1532–1542. 10.1037/dev0001529, 37166865 PMC10524449

